# Effects of Stimulus Rate and Periodicity on Auditory Cortical Entrainment to Continuous Sounds

**DOI:** 10.1523/ENEURO.0027-23.2024

**Published:** 2024-03-01

**Authors:** Sara Momtaz, Gavin M. Bidelman

**Affiliations:** ^1^School of Communication Sciences & Disorders, University of Memphis, Memphis, Tennessee 38152; ^2^Boys Town National Research Hospital, Boys Town, Nebraska 68131; ^3^Department of Speech, Language and Hearing Sciences, Indiana University, Bloomington, Indiana 47408; ^4^Program in Neuroscience, Indiana University, Bloomington, Indiana 47405

**Keywords:** auditory evoked potentials, auditory neural oscillations, periodicity coding, rhythmicity, time-frequency processing

## Abstract

The neural mechanisms underlying the exogenous coding and neural entrainment to repetitive auditory stimuli have seen a recent surge of interest. However, few studies have characterized how parametric changes in stimulus presentation alter entrained responses. We examined the degree to which the brain entrains to repeated speech (i.e., /ba/) and nonspeech (i.e., click) sounds using phase-locking value (PLV) analysis applied to multichannel human electroencephalogram (EEG) data. Passive cortico-acoustic tracking was investigated in *N* = 24 normal young adults utilizing EEG source analyses that isolated neural activity stemming from both auditory temporal cortices. We parametrically manipulated the rate and periodicity of repetitive, continuous speech and click stimuli to investigate how speed and jitter in ongoing sound streams affect oscillatory entrainment. Neuronal synchronization to speech was enhanced at 4.5 Hz (the putative universal rate of speech) and showed a differential pattern to that of clicks, particularly at higher rates. PLV to speech decreased with increasing jitter but remained superior to clicks. Surprisingly, PLV entrainment to clicks was invariant to periodicity manipulations. Our findings provide evidence that the brain's neural entrainment to complex sounds is enhanced and more sensitized when processing speech-like stimuli, even at the syllable level, relative to nonspeech sounds. The fact that this specialization is apparent even under passive listening suggests a priority of the auditory system for synchronizing to behaviorally relevant signals.

## Significance Statement

To examine the effects of stimulus factors on auditory cortical entrainment, we compared cortico-acoustic tracking for speech versus click stimuli across various speeds (below, at, and above the nominal speech rate) and acoustic periodicity (jitter in 20% steps). Overall, our results demonstrate that brain responses are more sensitive to changes in rhythm and periodicity of speech even during passive listening. The prioritization of speech in the brain might be partially due to the increased neuronal entrainment it elicits.

## Introduction

Temporal processing is a crucial component of audition (Picton, 2013) that influences all levels of auditory skills ranging from sensory levels to higher cognitive processing including attention and memory (Toplak et al., 2006; Allman and Meck, 2011). Indeed, the ability to time synchronize (i.e., entrain) to ongoing sound stimuli might represent a fundamental coding mechanism to support a variety of perceptual–cognitive processes including predictive coding and speech segmentation (Arnal and Giraud, 2012). In the broadest sense, entrainment refers to the synchronization (i.e., phase coupling) between two signals (Cummins, 2009). Such rhythmic fluctuations in terms of brain-to-stimulus coupling are characterized by excitation–inhibition cycles of neuronal populations termed “neuronal oscillations” (Bishop, 1932; Buzsaki and Draguhn, 2004; Lakatos et al., 2005; Buzsaki, 2006). In the context of speech, oscillatory neural entrainment is the process by which auditory cortical activity precisely tracks and adjusts to modulations in the speech amplitude envelope via phase alignment between neural and acoustic signals (Pikovsky et al., 2002; Lakatos et al., 2019). Entrainment is one of several important functions in auditory processing that can make communication seem effortless and automatic to healthy listeners and conversely, difficult in individuals with language learning disorders (Momtaz et al., 2021, 2022).

Entrainment applies to a wide range of physiologically important behaviors including auditory–motor coupling (Fujii and Schlaug, 2013; Herbst and Landau, 2016; Nozaradan et al., 2016; Moumdjian et al., 2018; Rosso et al., 2021) as well as speech (Peelle and Davis, 2012) and music (Fiveash et al., 2021) perception. For example, listeners must segment the continuous input speech signal into proper discrete units (i.e., syllables), which are then used as input for future decoding stages by auditory and nonauditory brain regions. In speech, the amplitude envelope's rhythmic information reflects different aspects of sensory and motor processing such as segmentation, speech rate, and articulation place and manner (Peelle and Davis, 2012). Moreover, the perceptual system recovers these rhythmic structures in speech, which are important for spoken language comprehension (Poeppel and Assaneo, 2020). Sensory processing presumably benefits from neural entrainment as it could provide a temporal prediction mechanism to anticipate future auditory events before they arrive at the ear (Lakatos et al., 2013).

On the contrary, auditory signals like speech might challenge an entrained system given its quasi-rhythmic temporal structure (Levelt, 1993; Guenther, 2016) that fluctuates with a speaker's talking rate. Still, speech production imparts temporal regularity to the signal envelope that is, on average, remarkably consistent in speed across the world's languages (Poeppel and Assaneo, 2020). Indeed, the temporal syllabic rate of speech across various languages ranges from 2 to 8 Hz with a “characteristic” periodicity of 4.5 Hz (Poeppel and Assaneo, 2020). It has been hypothesized that this range of rhythmicity is prioritized for speech perception and production (Assaneo and Poeppel, 2018). Moreover, speech intelligibility is optimal when the rhythmic structure of the signal falls inside a syllabic rate of 4–8 Hz (Peelle et al., 2013; Doelling et al., 2014). Thus, in addition to various speeds of representation, the quasi-periodic nature of speech effectively produces jitter which presumably also impacts entrainment and subsequent auditory processing.

Prior work has not fully elucidated how and to what extent aperiodicity might affect auditory processing (Doelling and Poeppel, 2015; Breska and Deouell, 2017; Novembre and Iannetti, 2018). Some studies demonstrate comparable neuronal phase-locking patterns for both periodic and aperiodic nonspeech stimuli (Wilsch et al., 2015; Morillon et al., 2016; Breska and Deouell, 2017). Nonetheless, upcoming events in speech can be anticipated by nonperiodic cues, for example, based on syntactic or semantic features (Nguyen et al., 2015). The predictability that results from periodic stimuli can also facilitate auditory perception and learning (Falk et al., 2017; Rimmele et al., 2018). Therefore, periodicity might facilitate auditory processing by providing temporal *predictability* to the system (Hovsepyan et al., 2020). At the very least, the brain must remain flexible and continuously adjust to changes in signal (a)periodicity to maintain robust processing. Yet, how different types of stimuli and the degree to which their aperiodicity affects auditory neural coding and entrainment remains unclear.

In the present study, we aimed to characterize how (1) rate, (2) periodicity, and (3) stimulus domain (i.e., speech vs nonspeech) affect auditory neural entrainment. In passive listening paradigms, we recorded multichannel EEGs in young, normal-hearing adults to assess neural entrainment to rapid auditory stimuli that parametrically varied in their speed (rate) and periodicity (temporal jitter). We analyzed the data at the source level to assess possible differences in hemispheric lateralization for entrained neural responses. The pacing of our rate manipulation assessed changes in neural oscillation strength for sounds presented slower than, at, and faster than the nominal syllabic rate of typical speech (i.e., 4.5 Hz). We reasoned that characterizing phase-locking strength across rates may demonstrate a preferred entrainment frequency of the system relative to rates that are considered to have special importance for speech perception. As a second manipulation, we evaluated the effects of signal (a)periodicity on entrained brain activity. By adjusting the successive interstimulus interval between repeated tokens, we varied the stimulus delivery between fully aperiodic and periodic presentation. As a third aim, we assessed the domain specificity of auditory neural entrainment. Studies using unintelligible sounds (Howard and Poeppel, 2010) have raised questions about whether brain entrainment mechanisms reflect mere physical stimulus characteristics (Capilla et al., 2011) or higher-level functions unique to speech-language processing (Peelle and Davis, 2012). Thus, in addition to speech, we mapped rate and jitter functions for nonspeech (click) stimuli to test for possible domain specificity in entrainment strength. Neural responses were then compared with standard psychoacoustical assays of rate and periodicity sensitivity to assess the behavioral relevance of our EEG findings.

## Materials and Methods

### Participants

We recruited *N* = 24 young adults (aged 20–39 years; 12 female, 12 male) to participate in the study. All participants had no history of neuropsychiatric illness and had normal hearing (i.e., air conduction thresholds ≤25 dB HL, screened from 500 to 4,000 Hz; octave frequencies). History of music training, years of education, and handedness were documented. We required participants to have <3 years of formal musical training since musicianship is known to enhance oscillatory EEG responses (Trainor et al., 2009; Bidelman, 2017). All participants were monolingual English speakers and were right-handed (mean score at the Edinburgh Handedness Inventory, 79; Oldfield, 1971). Participants gave written informed consent in compliance with a protocol approved by the Institutional Review Board at the University of Memphis (#2370) and were monetarily compensated for their time.

### Behavioral tasks and procedure

We used TMTFs and the CA-BAT paradigm (Viemeister, 1979; Bidelman et al., 2015; Harrison and Müllensiefen, 2018) to assess listeners’ perceptual rate and periodicity sensitivity and relate our neural findings to the behavior.

#### Temporal modulation transfer functions (TMTFs)

TMTFs are generally performed by modulating a carrier signal (e.g., noise) with a sinusoid at various rates and measuring the threshold modulation amplitude. The TMTF is a psychoacoustic measure of listeners’ sensitivity to track amplitude modulations. The TMTF function describes amplitude detection thresholds (i.e., absolute sensitivity) as a function of modulation frequency (Viemeister, 1973, 1979; Dau et al., 1997; Bidelman et al., 2015). TMTFs were measured using a forced-choice, adaptive tracking task. Three consecutive 500 ms bursts of wide-band noise (100–10,000 Hz) with 300 ms interstimulus interval (ISI) and 25 ms rise/fall ramping were presented binaurally using circumaural headphones (Sennheiser HD 280 Pro). The noise was set at 74 dB SPL. The first and third noise bursts had no modulation; the second burst was modulated with a sinusoidal envelope at rates of 2.1, 3.3, 4.5, 8.5, and 14.9 Hz, identical to those used for the EEG recordings. Participants adjusted the degree of modulation imposed on the noise so that the fluctuation in the second noise burst was just detectable. Plotting the minimum detectable modulation depth across various carrier frequencies (rates) gives the TMTF. Participants were allowed to adjust the modulation depth (measured in dB) using a slider bar on the computer screen until the difference between the target (modulated) and reference (unmodulated) intervals were no longer audible. The threshold was taken as the smallest modulation depth needed to just detect amplitude fluctuations in the stimulus. More negative thresholds reflect better task performance. This was repeated across rates to measure thresholds as a function of frequency. TMTFs were measured using the Auditory Interactivities Software (Sensimetrics Corp., Gloucester, MA).

#### CA-BAT

The computerized adaptive beat alignment test is a version of the beat alignment test that assessed participants’ behavioral sensitivity to periodicity, that is, jitter (Harrison and Müllensiefen, 2018). The test consisted of 27 items lasting ∼10 min that were presented with an intensity of ∼74 dB SPL. Each item consisted of a beep track superimposed on a musical clip. The beep track alignment (*d_r_*) varied adaptively from trial to trial (0.5 ≤ *d_r_*_ _< 1) such that it was displaced in the direction ahead or behind the music. Increasing *d_r_* moved the beep track closer to the musical beat and made discrimination harder. Critically, *d_r_* was varied adaptively based on the listener's trial-to-trial performance to converge onto their threshold for periodicity sensitivity. Participants were provided some sample music before the testing session as a training phase that includes instructions, audio demonstrations, and two practice questions. They were then given the 27 musical track test items in random order during the data collection phase, with no item-to-item feedback. The task was a two-alternative forced-choice (2-AFC) paradigm. On each trial, listeners heard two versions of the same musical track; they differed only in the overlaid metronome beep track. In one interval, the metronome and music were synchronized. In the other (lure) interval, they were displaced by a constant proportion of a beat. Participants were instructed to choose the one that was synchronized. The main output from the CA-BAT is an *ability score* (range, −4 to 4), corresponding to the listener's sensitivity to periodicity. A secondary output is an *ability_sem score*, corresponding to the standard error of measurement for the ability estimate. Both metrics are computed from the underlying item response theory model (Harrison and Müllensiefen, 2018). The paradigm was implemented in R (v.4.1.1; R Core Team, 2013).

### EEG recording procedures

#### Stimuli

**Figure 1.**
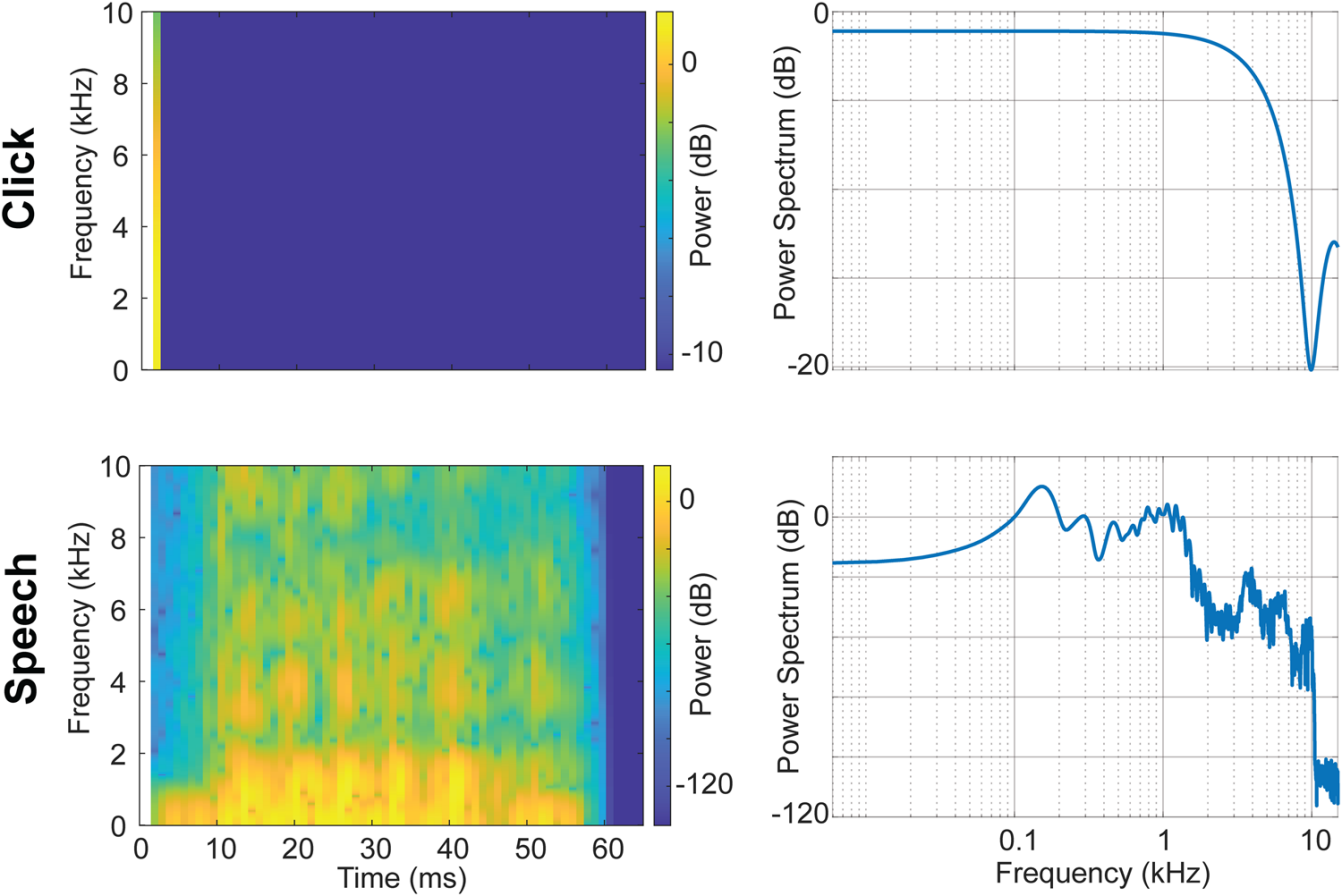
Spectral properties of the isolated click and speech /ba/ tokens. First column, Stimulus spectrograms and right column, power spectra. Note the similarity in overall bandwidth of the speech and nonspeech stimulus. Level is expressed in arbitrary units to show the relative changes in dB across the power spectrum of the isolated tokens (apparent overall level differences are arbitrary before calibration). For actual stimulus presentation, isolated tokens were strung together to produce trains of stimuli which were then equated in RMS amplitude. Thus, despite clear variation in their power spectra, overall perceptual loudness was similar between speech and click *trains* (see also footnotes #1 and #2).

EEGs were elicited using trains of clicks (100 µs) and the synthesized speech (60 ms) token /ba/ (Fig. 1). Click and speech tokens were matched in overall level and bandwidth. Acoustic stimuli were presented at a sampling rate of 48,818 Hz to ensure maximal acoustic bandwidth during stimulus presentation. The speech token was selected as pilot testing determined it was the most identifiable token among several consonant–vowel options from previous neural oscillation studies (/ma/, /wa/, /va/, and /ba/; Assaneo and Poeppel, 2018). In the rate experiment, click and /ba/ tokens were presented at five different rates (2.1, 3.3, 4.5, 8.5, 14.9 Hz).

**Figure 2.**
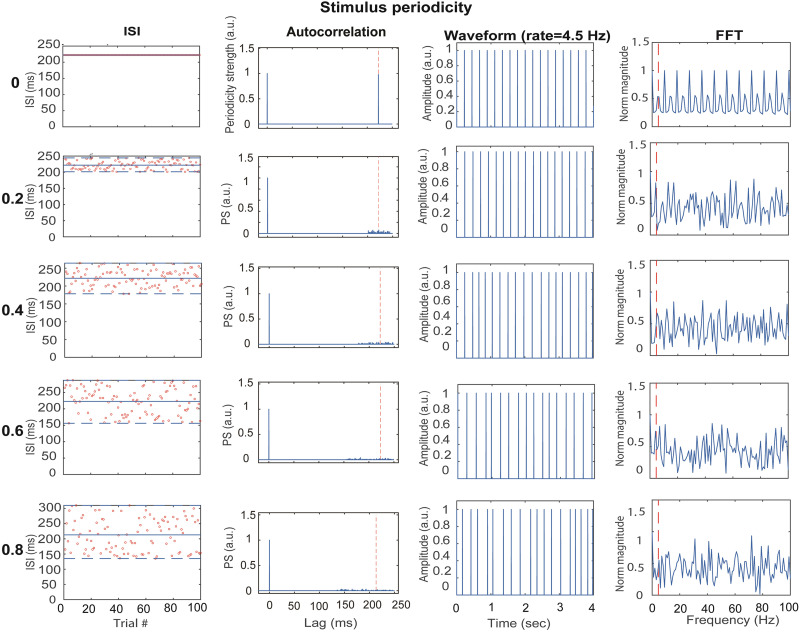
Acoustic properties illustrating the effects of (a)periodicity jitter (all clicks at 4.5 Hz rate; nominal ISI = 1/4.5 Hz = 222 ms). First column, Distribution of ISI around the nominal ISI as a function of jitter from 0 to 80%. Second column, Autocorrelation functions (ACFs) show the degree of periodicity in the stimuli. Note the periodicities at 222 ms which becomes blurred around the nominal period (dotted lines) with increasing jitter. Third column, Time waveforms. Fourth column, Fast Fourier transforms (FFTs) as a function of jitter. As with the ACFs, note the decreased energy at 4.5 Hz (dotted lines) with increasing stimulus jitter/aperiodicity.

In the jitter experiment, this token was presented at the nominal syllabic rate of speech (4.5/s), but we varied the trains’ periodicity by introducing random jitter in half the ISI between successive tokens. Jitter ranged from perfectly periodic (nominal ISI/2 ± 0% jitter) to aperiodic trains (nominal ISI/2 ± 80% jitter) in five equal steps from 0 to 80% (20% steps; Krumbholz et al., 2003). Importantly, ISIs were uniformly sampled around the nominal rate (222 ms = 1/4.5) which maintained the overall average rate of stimuli between periodic and aperiodic conditions, allowing only the degree of periodicity to vary (Fig. 2). Both click and speech stimuli were presented binaurally at 74.3 dB SPL via ER-2 insert earphones (Etymotic Research). Stimulus level was calibrated using a Larson–Davis SPL meter (Model LxT) measured in a 2 cc coupler (IEC 60126) (Stimulus level was equated using recommended procedures for calibrating transient stimuli (ISO389-6, 2007). Click/speech tokens were presented in a continuous train at the fastest rate (14.9 Hz). The resulting steady-state RMS was then adjusted to achieve 74.3 dB SPL for both speech and nonspeech stimuli to match overall level and thus perceptual loudness (see also footnote #2)). Left and right ear channels were calibrated separately. The study encompassed both speech and click conditions, involving a total of five rates (2.1, 3.3, 4.5, 8.5, 14.9 Hz) and five jitter conditions specifically applied at the 4.5 Hz rate (0, 20, 40, 60, 80%). In total, there were 18 distinct conditions (the 0% jitter was the same in the 4.5 Hz rate). Each condition consisted of 1,000 tokens. All conditions were randomized for each participant.

#### EEG recording

Participants were seated in an electrically shielded, sound-attenuating booth during EEG testing. They were asked to keep their eyes open and watch a silent selected movie (i.e., passive listening task). Passive listening allows for the investigation of spontaneous neural responses without the influence of specific cognitive or attentional demands. We use a passive task here to explicitly test whether previously reported enhancements in neural speech tracking near the language-universal syllable rate (4–5 Hz) depend on attention (Assaneo and Poeppel, 2018; Assaneo et al., 2019a; He et al., 2023b) or instead reflect more automatic tuning of auditory entrainment processing. Continuous EEGs were recorded using a 64-channel electrode cap (Neuroscan Quik-Cap). Blink artifacts were monitored by placing electrodes on the outer canthi and superior and inferior orbit of the eyes. Electrode positions in the array followed the international 10–10 system (Oostenveld and Praamstra, 2001). Electrodes were maintained at <5 kΩ impedance during testing and were rehydrated halfway through the experiment as necessary. EEGs were recorded using Neuroscan SynAmps RT amplifiers at a sample rate of 500 Hz. Data were re-referenced to the common average offline for subsequent analysis.

### EEG analysis

We used BESA Research 7.1 (BESA) to transform each listener's single-trial scalp data into source space using BESA's auditory evoked potential (AEP) virtual source montage (Scherg et al., 2002; Bidelman, 2017). This applied a spatial filter to all electrodes that calculate their weighted contribution to the scalp recordings. We used a four-shell spherical volume conductor head model (Sarvas, 1987; Berg and Scherg, 1994) with relative conductivities (1/Ωm) of 0.33, 0.33, 0.0042, and 1 for the head, scalp, skull, and cerebrospinal fluid, respectively, and compartment sizes of 85 mm (radius), 6 mm (thickness), 7 mm (thickness), and 1 mm (thickness; Picton et al., 1999; Herdman et al., 2002). The AEP model includes 11 regional dipoles distributed across the brain including bilateral auditory cortex [AC; Talairach coordinates (*x*, *y*, *z*; in mm): left (−37, −18, 17) and right (37, −18, 17)]. Regional sources consist of dipoles describing current flow (units nAm) in tangential planes. We extracted the time courses of the tangential components for left and right AC sources as this orientation captures the majority of variance describing the auditory cortical ERPs (Picton et al., 1999). This approach allowed us to reduce each listener's 64-channel data to two source channels describing neuronal currents localized to the left and right AC (Price et al., 2019; Momtaz et al., 2021).

#### Phase-locking value (PLV)

We computed phase-locking value (PLV; Lachaux et al., 1999) between brain and stimulus waveforms to evaluate how neural oscillatory responses track speech and nonspeech acoustic signals across different presentation speeds (rates) and periodicities (jitter). This allowed us to assess neuro-acoustic synchronization across tokens in the ongoing sound stream. We first transformed the continuous EEGs into source waveforms (SWFs) via matrix multiplication of the sensor data (EEG) with the AEP source montage's dipole leadfield (L) matrix (i.e., SWF = *L*^−1 ^× EEG; Scherg et al., 2002; Bidelman, 2018). The leadfield was based on the dipole configuration detailed above. This resulted in two time series representing current waveforms in source space projected from left and right AC. For submission to PLV analysis, 30 s of continuous data was then extracted. Importantly, this yielded equal-length neural data per stimulus condition and listener. Identical processing was then applied to all rate and jitter conditions per participant.

We measured brain-to-stimulus synchronization as a function of frequency via PLV (Lachaux et al., 1999). First, we computed the full-band envelope from the stimulus via the Hilbert transform. We then down-sampled the stimulus envelope to match the sampling rate of the EEG (i.e., 500 Hz). To be reasonably interpreted, PLV requires bandpass filtering the signals to assess how entrainment changes in a frequency-dependent manner. Following approaches by Assaneo and Poeppel (2018) and He et al. (2023a,b), neural and acoustic stimulus signals were bandpass filtered (±0.5 Hz) around each nominal frequency, and PLV was then computed according to Eq. 1:

(1) $$\mathrm{PLV}=\frac{1}{T}\left|\overset{T}{\underset{t=1}{\sum }}\;e^{i[\theta_{1}(t)-\theta_{2}(t)]}\right|,$$

where *θ*_1_(*t*) and *θ*_2_(*t*) are the Hilbert phases of the EEG and corresponding evoking stimulus signal, respectively. Intuitively, PLV describes the average phase difference (and by reciprocal, the correspondence) between the two signals. PLV ranges from 0 to 1, where 0 represents no (random) phase synchrony and 1 reflects perfect phase synchrony between signals. We then repeated this procedure—that is, isolating a 1 Hz band and computing PLV—for center frequencies between 1.1 and 30 Hz (0.3 Hz steps). This resulted in a continuous function of PLV describing the degree of brain-to-stimulus synchronization across the bandwidth of interest (Assaneo et al., 2019b; Fig. 4). We then measured PLV magnitude for each rate/jitter, stimulus type (speech vs click), and participant. The magnitude was taken as the peak of each individual frequency-dependent PLV function within ±0.5 Hz of the nominal stimulus rate (Fig. 4, ▾s; He et al., 2023a,b). Comparing PLV magnitude across increasing rates/jitters allowed us to characterize how brain-to-stimulus synchronization varied for speech versus nonspeech stimuli and between cerebral hemispheres. However, the omnibus ANOVA on PLV measures failed to reveal main or interaction effects with hemisphere (results reported below). Consequently, we collapsed LH and RH responses to focus on variations in peak PLV across stimulus manipulations (i.e., rates and jitters).

### Statistical analysis

Unless otherwise noted, we used mixed-model ANOVAs implemented in R (lme4 package;(Bates et al., 2014) to assess all dependent variables of interest. Fixed factors were rate (2.1, 3.3, 4.5, 8.5, 14.9 Hz), periodicity (0, 20, 40, 60, 80% jitter), and stimulus domain (click, /ba/). Subjects served as a random effect. Based on the distribution of the data and initial diagnostics, we transformed the data using a square root transformation. The significance level was set at *α* = 0.05. Tukey–Kramer adjustments were used for post hoc contrasts. Correlations (Pearson's *r*) were used to evaluate relationships between neural oscillations and behavior.

## Results

### Behavioral data

#### TMTFs (rate sensitivity)

**Figure 3.**
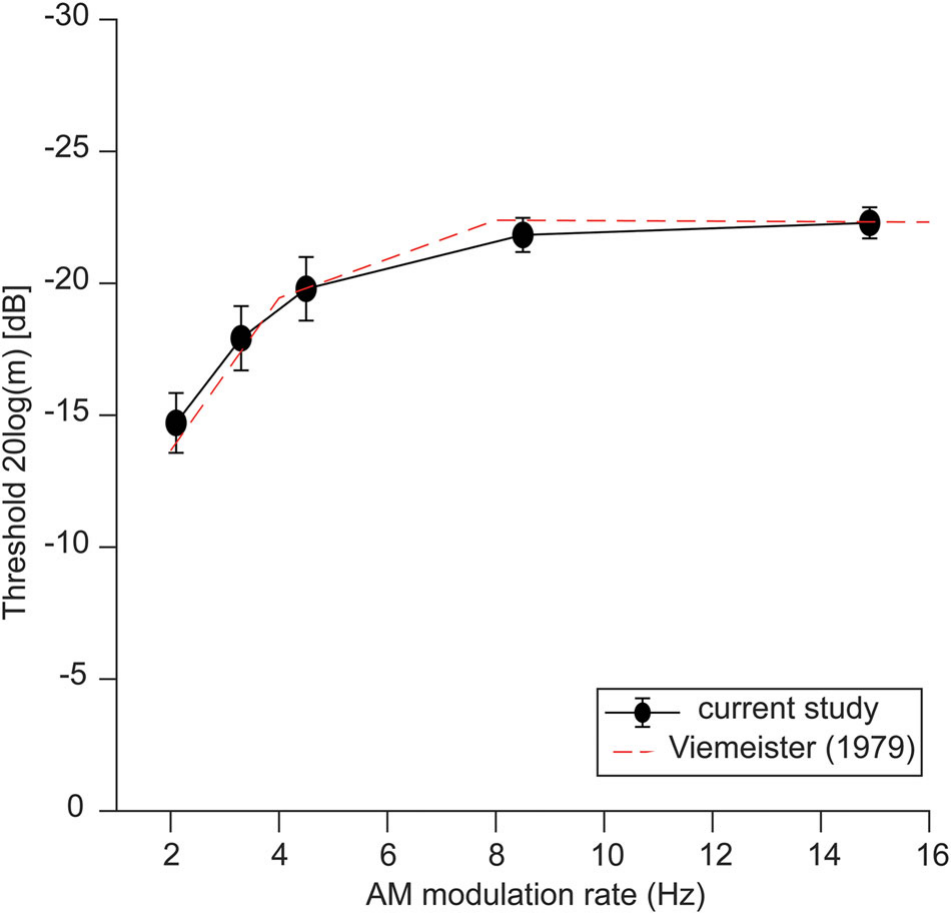
TMTFs show behavioral rate sensitivity. TMTFs demonstrate temporal acuity for detecting AM fluctuations in sounds as a function of modulation frequency. For very low frequencies <20 Hz, and consistent with Viemeister (1979; see 500 ms gated carrier condition, their Fig. 6), TMTFs demonstrate a high-pass filter shape, indicating slight improvements in behavioral rate sensitivity from 2 to 20 Hz. Error bars = ±1 SEM.

Figure 3 shows the average TMTFs of our participants and the data of Viemeister (1979) for comparison. TMTFs show sensitivity (threshold) to amplitude modulation in wide-band noise measured at five different rates. With increasing rates, participants showed better (i.e., more negative) detection thresholds corresponding to better sensitivity (i.e., temporal resolution). An ANOVA on TMTF thresholds revealed a rate effect on TMTF thresholds (*F*_(4,115)_ = 10.44; *p* < 0.001). TMTF thresholds typically worsen with increasing rates up to ∼100 Hz. However, at the low modulation rates used in this study—and consistent with prior psychoacoustic studies (Viemeister, 1979)—we find that rate sensitivity increases slightly between 2.1 and 14.9 Hz.

#### CA-BAT (aperiodicity sensitivity)

The CA-BAT produced two different scores of periodicity sensitivity for each participant related to the absolute threshold (ability score) and its variance (ability_sem; Harrison and Müllensiefen, 2018). Ability scores averaged 0.17 ± 0.92 across participants, consistent with prior psychoacoustic studies on jitter sensitivity (Harrison and Müllensiefen, 2018).

### EEG oscillations across token (PLV)

**Figure 4.**
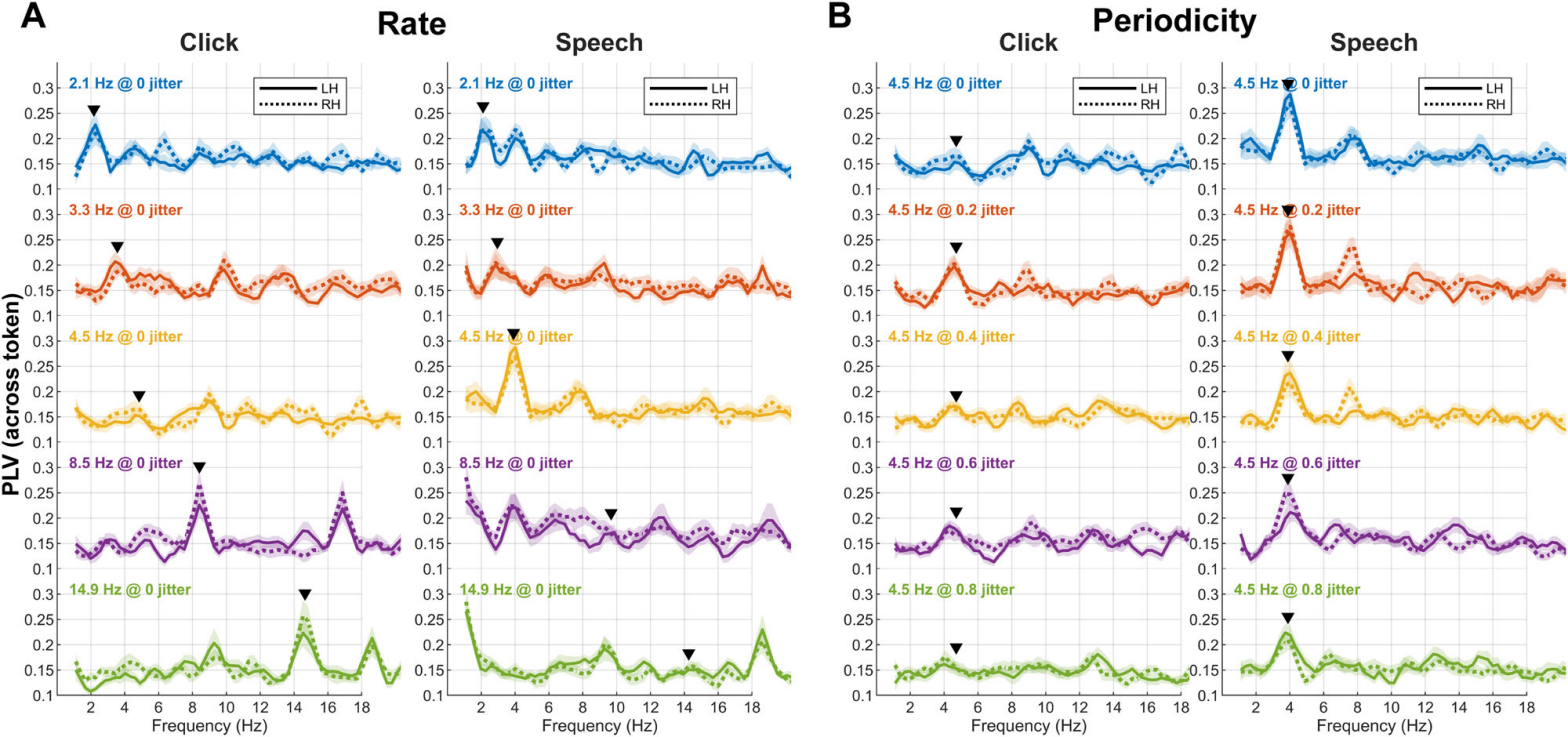
Grand average PLV across subjects as a function of rate, jitter, stimulus, and hemisphere. ***A***, Rate results. ***B***, Periodicity results. PLV showed enhanced activity at each fundamental frequency (▾) and integer-related harmonics. The right and left hemispheres exhibited comparable responses. Shading = ±1 SEM.

**Figure 5.**
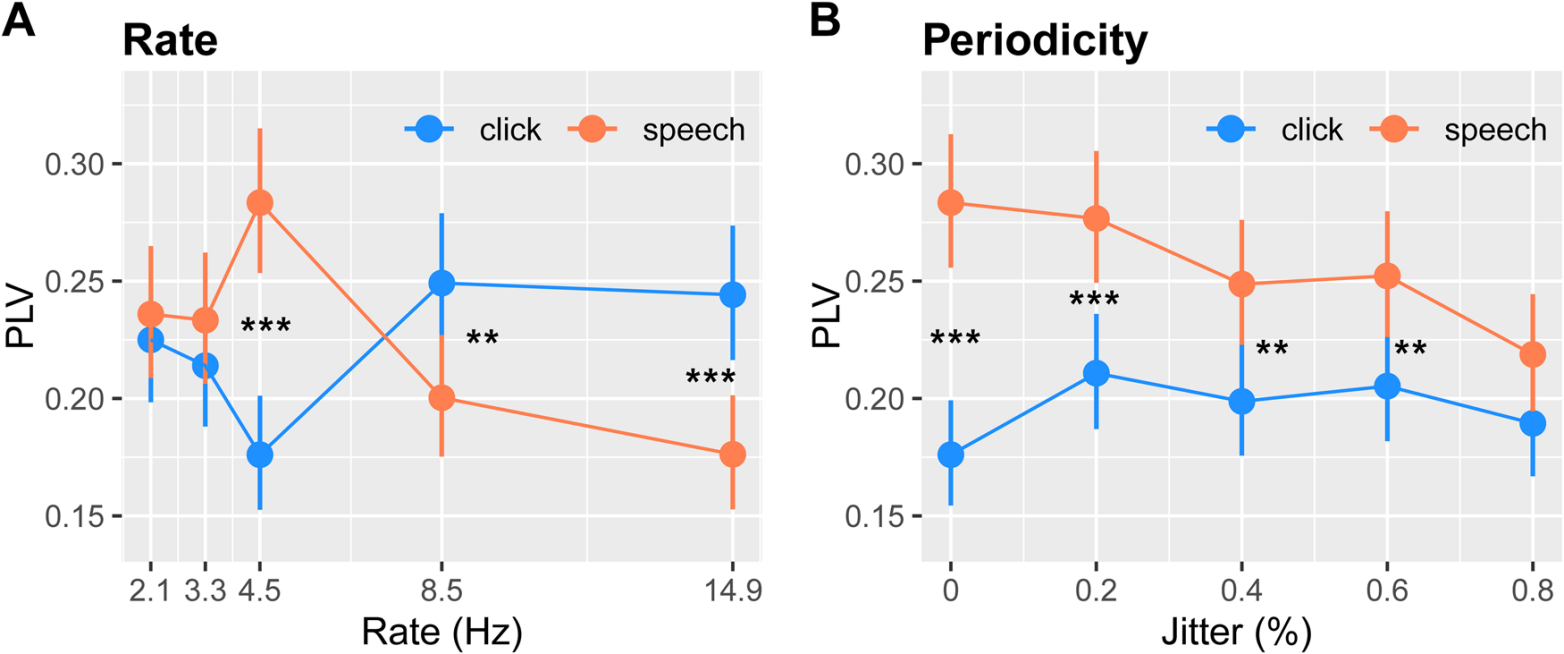
Grand average peak PLV across rate and jitter (aperiodicity). ***A***, Rate effects show a rate × stimulus domain interaction. The speech token /ba/ elicited increased neural entrainment at 4.5 Hz. ***B***, Periodicity effects show a stimulus domain × jitter interaction. Neural entrainment to speech is initially enhanced compared with clicks at low jitters but declines within increasing aperiodicity. PLV strength is invariant to increasing jitter for clicks. PLV, phase-locking value. Responses are collapsed across hemispheres. Error bars = ±95% CI. *p < 0.05, **p < 0.01, ***p < 0.001. See also Extended Data Fig. 5-1.

We used PLV to quantify neural phase locking across tokens and how the brain entrains the ongoing stream of acoustic stimuli. Raw PLV response functions illustrating changes in phase-locking strength as a function of frequency, stimulus manipulations (rate, jitter), hemispheres (RH, LH), and stimulus type (click, /ba/) are shown in Figure 4. Peak quantification of the PLV functions is shown in Figure 5. In general, neural responses closely followed the speed of the auditory stimuli, showing stark increases in PLV strength that closely followed the fundamental rate of presentation (i.e., *F*0 = 2.1–14.9 Hz). Harmonics were also observed, which are common in the spectra of sustained auditory potentials and are due to nonlinearities of the EEG that result in phase locking at the *F*0 and its integer multiples (2*F*0, 3*F*0, …, *nF*0; Lins et al., 1995; Bidelman and Bhagat, 2016). PLV strength also varied for the 4.5 Hz stream with changes in jitter; more aperiodic sounds produced weaker neural entrainment.

10.1523/ENEURO.0027-23.2024.f5-1 Figure 5-1 **Grand average peak stimulus-to-EEG cross-correlation across (A) rate and (B) jitter aperiodicity.** Cross-correlations highly differ from the PLV pattern observed for rate. They partially replicate the PLV analysis in Fig. 5 for jitter but do not show a jitter* stimulus interaction like PLV. Errorbars denote 95% CIs. Download Figure 5-1, TIF file.

**Figure 6.**
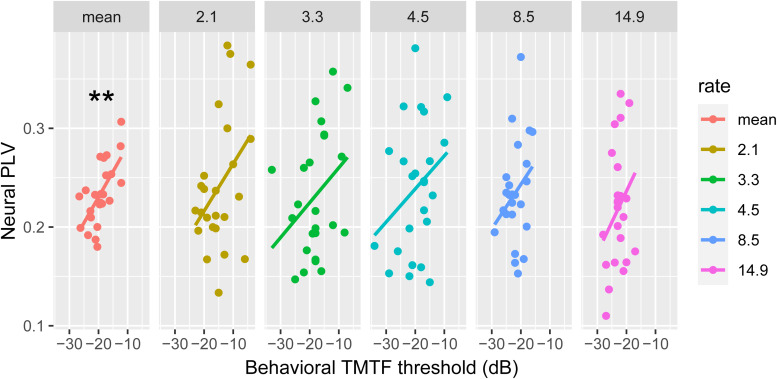
Brain–behavioral correlations for rate sensitivity. Behavioral TMTF thresholds show an association with the mean PLV averaged across rates (**p < 0.01). Values are pooled across speech/click conditions and hemispheres. Scatters show brain–behavior correlations between TMTF thresholds and the five individual rates (all n.s. by themselves).

An ANOVA assessing rate effects on peak PLV showed a rate × stimulus domain interaction (*F*_(4,437) _= 13.64; *p *< 0.0001; Fig. 5*A*). Multiple comparisons showed stronger PLV for speech versus clicks at 4.5 Hz (*p *< 0.0001). This pattern reversed at higher rates with speech eliciting lower PLV than clicks at 8.5 (*p *= 0.0095) and 14.9 Hz (*p *= 0.0002). These results suggest neural entrainment for speech stimuli is enhanced relative to nonspeech signals specifically at 4.5 Hz and weakens precipitously for higher rates beyond what typically occurs in normal speech production.

For periodicity, we found a main effect of stimulus domain (*F*_(1,437) _= 67.75; *p *< 0.0001), but more importantly, a periodicity × stimulus domain interaction (*F*_(4,437) _= 3.18; *p *= 0.013; Fig. 5*B*). The main effect of stimulus was due to speech (all 4.5 Hz rate) eliciting stronger PLV than clicks across the board. Indeed, individual click versus speech contrasts at each jitter revealed higher PLV for speech than clicks that diminished with increasing aperiodicity: 0% (*p *< 0.0001), 20% (*p *= 0.001), 40% (*p *= 0.0021), 60% (*p *= 0.0042), and 80% (*p *= 0.058). In other words, the overall pattern for speech exhibited a linear decline in PLV (linear effect; *t*_437 _= −4.01; *p *< 0.0001) where the strength decreased with increasing jitter. In contrast, entrainment to clicks remained constant with increasing jitter (linear effect; *t*_(437) _= 0.66; *p *= 0.51). Indeed, when compared directly, the linear trend effect was stronger for speech than clicks (*t*_(437) _= −3.299; *p *= 0.0011). This interaction suggests that speech produces stronger neural entrainment across tokens than nonspeech sounds and is also more impervious to disruptions in periodicity (i.e., signal jitter).

One concern is that the increased jitter of a signal spreads the power across a wider frequency range and conversely, lessens the power in a narrow frequency band near the *F*0 rate. This might artificially reduce PLV with increasing aperiodicity. To test this possibility, we computed cross-correlations (MATLAB xcorr function) between the stimulus acoustic envelope waveforms and the EEG (Extended Data Fig. 5-1). In general, cross-correlation effects mirrored but were less salient than PLV effects. An ANOVA conducted on cross-correlations revealed only main effects of jitter (*F*_(4,437) _= 4.64; *p *= 0.0011) and stimulus domain (*F*_(1,437) _= 15.32; *p *< 0.0001). Critically, however, we did not find a jitter × stimulus interaction (*F*_(4,437) _= 0.76; *p *= 0.55) as observed for PLV. Similarly, cross-correlations across rate showed a different pattern than for PLV. While there was a rate (*F*_(4,437) _= 5.18; *p *= 0.0004) and stimulus effect (*F*_(1,437) _= 12.19; *p *= 0.0005), there again was no rate × stimulus interaction (*F*_(4,437) _= 1.21; *p *= 0.31) as observed for PLV. These findings suggest that while the gross pattern of PLV effects observed in the data (Fig. 5) might be partially accounted for changes in stimulus-to-response correlation with increasing jitter, PLV reveals an additional *differential* sensitivity in neural phase-locked entrainment between speech and nonspeech stimuli.

### Brain–behavior relationships

We used correlations to explore the correspondence between neural responses and behavioral measures. The average PLV across rates was highly correlated with TMTF thresholds (*r* = 0.65; *p* = 0.0006); larger neural PLV was associated with poorer (less negative) behavioral thresholds. The correlations for each individual rate were not significant at 2.1 Hz (*r* = 0.39; *p* = 0.06), 3.3 Hz (*r* = 0.33; *p* = 0.11), 4.5 Hz (*r* = 0.29; *p* = 0.16), 8.5 Hz (*r* = 0.28; *p* = 0.19), or 14.9 Hz (*r* = 0.28; *p* = 0.19). For the CA-BAT test, the degree of periodicity sensitivity at 0.6 jitter was negatively correlated with PLV (*r* = −0.41; *p* = 0.044; data not shown). That is, better periodicity detection thresholds were associated with improved entrainment for ∼60% jittered stimuli. However, we note this correlation was weak and occurred only after poling across hemispheres and stimulus type. Still, these findings indicate participants’ behavioral sensitivity to periodicity was loosely associated with their entrained brain responses.

## Discussion

We compared cortico-acoustic tracking for repeated speech versus click train stimuli parameterized across various speeds (below, at, and above the nominal speech rate) and degrees of acoustic periodicity (jitter in 20% steps) to probe the effects of these stimulus factors and map the profiles of oscillatory activity for auditory cortical entrainment. For *rate*, we found that phase locking to the repetitive speech token /ba/ showed a surprising improvement in neural entrainment at 4.5 Hz but deteriorated at higher rates. For clicks, however, phase-locking strength dropped at 4.5 Hz and then rebounded sharply at 8.5 Hz until peaking at 14.9 Hz. For *periodicity*, we found that while phase locking to speech declined with increasing jitter, entrainment to speech was still superior to that of clicks. In contrast to /ba/ sounds, click responses were largely resistant to disruptions in periodicity. Collectively, our findings show that even under passive listening, auditory neural entrainment for speech relative to nonspeech sounds is (1) more sensitive to changes in rate and periodicity and (2) enhanced in strength, perhaps reflecting a prioritization for synchronizing to behaviorally relevant sounds.

### Rate effects

Continuously changing speech rate/context requires ongoing adjustment by the listener (Casas et al., 2021). Our stimulus design included five rates intending to examine the auditory system's temporal processing of speech and nonspeech sounds at speeds below, at, and above typical syllabic rates for speech (Poeppel and Assaneo, 2020). We found an interaction between rate and stimulus type for PLV measures. Slow theta waves easily follow the envelope of ongoing speech and are thus thought to integrate syllable representations across tokens—a larger level of the speech-analysis hierarchy (Casas et al., 2021). Our findings indicate that PLV exhibited a strong differentiation between repeated click and speech-syllable trains. PLV reflects the phase locking between the acoustic stimulus and EEG response across tokens and over the time course of the stimulus stream. Surprisingly, across-token PLV to speech revealed strong enhancements in phase locking at 4.5 Hz, validating the concept of a universal speech syllabic rate across global languages (Assaneo and Poeppel, 2018; He et al., 2023b). Our results here extend these prior studies, however, by demonstrating such enhanced synchronization at 4.5 Hz during passive listening, in the absence of an active speech perception task. Critically, our data also show that phase-locking enhancement at ∼4.5 Hz was specific to speech and did not occur for clicks (i.e., domain-specific effect). Moreover, we found phase locking to speech plummeted at higher frequencies, suggesting these speech-specific enhancements are limited to stimulus rates that occur naturally for speech (Assaneo and Poeppel, 2018).

It is difficult to see how duration effects alone could account for the differential (interaction) pattern observed in our data between speech and click trains. Indeed, there is some evidence that auditory cortical entrainment is enhanced for intelligible signals which might explain the larger PLV we see for the syllable train relative to click stimuli (Xu et al., 2023). Moreover, token duration seems to play a less prominent role in the strength of cortical entrainment since tracking is dominated by locking to an acoustic signal's edge landmarks rather than its nucleus, per se (Oganian and Chang, 2019; Oganian et al., 2023).

At higher rates, and in contrast to speech, click synchronization actually improved slightly. The counterintuitive enhancement of click responses at the higher rates is however consistent with psychoacoustical findings (including those here), explained as increased summation (i.e., temporal integration) of postsynaptic potentials as a result of activating a broader neural population (Forss et al., 1993; Brugge et al., 2009). Thus, while the auditory system is certainly capable of synchronizing to higher stimulus rates (as evidenced by our click data), it appears as though the sensitivity to modulations in speech synchronization is more restricted.

### Periodicity effects

Various temporal scales of the speech signal and linguistic hierarchy are correlated with neural entrainment in different frequency bands of brain activity (Ghitza and Greenberg, 2009). Given that speech is not perfectly periodic and shorter syllables frequently follow or precede longer ones, it is intriguing to examine how the (a) periodicity of speech influences brain entrainment (Ghitza and Greenberg, 2009). By parametrically varying the jitter of otherwise periodic signals, we aimed to perturb the input integrity and examine how brain rhythms to the optimal speech rate (at 4.5 Hz; Assaneo and Poeppel, 2018) are disrupted by aperiodicity. In classic evoked response paradigms, which only consider token-wise responses, the temporal information between successive stimulus events does not directly affect the neural representation of the acoustic signal, at least at lower rates where adaptation would be at play. However, other studies showed that “cross-token comparisons” could indeed be a cue for temporal coding that has a significant impact on intelligibility (Miller and Licklider, 1950; Huggins, 1975). Thus, temporal perturbation can affect how sounds are organized and subsequently perceived. Our PLV measurements robustly detect these jitter effects especially for speech events.

Periodicity in speech has been shown to facilitate perception and intelligibility (Benesty et al., 2008). Ghitza and Greenburg (2009) altered the interstimulus intervals between syllables using periodic and aperiodic interruption to affect intelligibility. Their results ascribed neural entrainment to internal processing rather than acoustic aspects of the sounds (Ghitza and Greenberg, 2009). They showed the speech intelligibility of the compressed signal is poorer than that of the original signal. In their study, inserting 20–120-ms-long gaps of silence into a speech significantly increases its intelligibility. Our study design excluded an intelligibility component as it was passive and focused arguably on only the acoustic-phonetic characteristics of speech. Still, our results reveal that neural entrainment is improved for rhythmic speech as opposed to click or even aperiodic speech. It is possible such neural effects account for the perceptual facilitation observed for periodic signals in previous behavioral studies (Ghitza and Greenburg, 2009).

Only the 4.5 Hz presentation rate was chosen in the jitter experiment; we anticipated optimal phase locking at this pace due to its alignment with the nominal syllable rate of most languages (Assaneo and Poeppel, 2018). PLV demonstrated that at this specialized rate, periodicity did not affect cortical entrainment to clicks. Despite the fact that clicks are more perceptually salient than speech due to their rapid onset (Vigil and Pinto, 2020), jitter did not affect PLV entrainment sensitivity for clicks. With increasing the jitter of speech stimuli, however, phase locking deteriorated. It is possible that as speech becomes more aperiodic, the brain treats the signal more like a nonspeech stimulus, resulting in similarly low PLV as we observe for click stimuli. This could explain why stimulus type and jitter interacted in our PLV analysis. We can rule out explanations of these effects due to loudness differences as all tokens were similar in overall SPL as well as perceptual loudness (96.9 ± 2.7 phons; Moore et al., 1997) (Loudness was computed using the MATLAB function acousticLoudness() according to standard ISO532-2 (2017). The similarity in (estimated) loudness across stimuli is perhaps unsurprising given that the RMS amplitude was equated across tokens (see Materials and Methods and footnote #1)). In fact, speech stimuli, which elicited more robust PLV, were actually ∼5 phons *weaker* in perceptual loudness than clicks, contrary to a temporal integration explanation of the data. On the contrary, periodicity in speech may boost neural responses via predictive coding, which could account for the stronger entrainment to speech we find at low relative to high degrees of jitter (Thut et al., 2011; Peelle and Davis, 2012). Previous research also suggests specific populations of neurons that respond to aperiodic but not periodic stimuli (Yrttiaho et al., 2008). Also, imaging studies demonstrate the location of periodicity-sensitive cortical areas can be more anterior than aperiodic stimuli, depending on the stimulus type (Hall and Plack, 2009). Our technique for EEG source analysis only localized responses in the auditory cortex, which may not elicit equivalent responses for speech and click stimuli (Steinschneider et al., 1998).

### Brain–behavior relations between entrainment and rate/periodicity sensitivity

We demonstrate an association between behavioral sensitivity and brain synchronization to stimuli varying in rates. Individuals who showed greater sensitivity in our psychoacoustic measures exhibited weaker phase locking in cortical entrainment. A recent study by Casas et al. (2021) also showed that participants that are more behaviorally responsive to temporal changes in ongoing sounds exhibit weaker phase-locked responses. They attributed their counterintuitive findings to the sample size and other methodological concerns including the perceptual difficulty of their stimulus set (Casas et al., 2021). However, one possibility is that their neural recordings included several entrained responses from multiple brain areas (not exclusively auditory regions). Indeed, conventional EEG suffers from volume conduction resulting in frontal generators contributing to auditory evoked responses (Knight et al., 1989; Picton et al., 1999). Our use of passively presented stimuli and source analysis helps exclude attentional or task confounds (as suggested by Casas et al., 2021) that are likely modulated by frontal cortical areas. Having the same result, however, it is possible that individuals who show better brain-to-acoustic coupling relegate temporal processing to lower levels of the auditory system (e.g., brainstem, thalamus), more peripheral to the behavior and cortical responses assessed here. Whereas others who perform worse or more laboriously in temporal processing tasks might require high levels of auditory processing at the cortical level as a form of compensatory mechanism (Momtaz et al., 2021, 2022). This might account for the counterintuitive negative correlation we find between cortical phase-locking strength and behavior for the rate manipulation, that is, more effortful encoding (higher PLV) in less perceptually sensitive individuals. Other electrophysiological TMTF studies reveal a decrease in neural sensitivity with increasing modulation rate (Wang et al., 2012). These results run counter to what we observe here and suggest differences in stimulus acoustics (e.g., bandwidth) and analysis techniques might account for discrepancies across studies. However, we did find that CA-BAT periodicity detection thresholds weakly predicted PLV strength for jitter manipulations (at least the ∼60% jitter). We note that a significant distinction between our brain and behavior data is that although TMTFs and CA-BAT measure rate modulation and jitter detection thresholds in active tasks, our neural recordings were conducted under strictly passive listening. It is highly likely the nature of the correlation would change had we conducted our behavioral tasks during the EEG recordings. Consequently, given our strictly passive listening paradigm, interpretations that our brain–behavioral correlations index listening effort remains speculative and should be confirmed in future studies.

In this vein, while our results here demonstrate periodic speech-like stimuli differentially affects neuronal entrainment in passive listening conditions, how these mechanisms might change in active attentional states remains unknown. Presumably, active tasks during entraining stimuli might also recruit additional (nonauditory) brain regions other than the auditory cortex, which was the focus of this investigation. As such, functional connectivity approaches (Rimmele et al., 2018) might be used to further tease out the dynamics of bottom-up (sensory-driven) versus top-down (cognition-driven) processing and interhemispheric connections that affect the brain's ability to entrain to rapid auditory stimuli.

## Conclusions

Overall, this study aimed to address questions regarding rate, periodicity, and stimulus differences in EEG neural entrainment and their association with behavioral responses. By examining the same parameters, our data reveal unique distinctions in how each of these factors impacts the neural encoding of ongoing complex sounds.

Our findings show that the brain's entrainment to repeated speech tokens (phonemic level processing) is rate and periodicity sensitive and more so than for nonspeech clicks. These findings might inform broader work examining temporal processing issues in patient populations such as those with certain auditory processing disorders (Momtaz et al., 2021, 2022) or dyslexia (Tallal et al., 1993; Ben-Yehudah and Ahissar, 2004) which impact auditory temporal processing. The data here helps characterize the constraints on temporal capabilities of auditory cortex and neural entrainment at early stages of speech perception. It would be interesting to extend the current paradigm to future studies in these clinical populations. Auditory plasticity induced by training and rehabilitative programs that aim to enhance temporal processing (Chermak and Musiek, 2002; Anderson and Kraus, 2013; Moreno and Bidelman, 2014) could be used to enhance cortical phase locking and, subsequently, speech understanding in challenging listening environments. The stimulus specificity we observe in entrainment patterns also speaks to the need to incorporate the correct stimuli in training plans if the goal is to maximize neural and behavioral outcomes. Indeed, our data suggest periodic speech at or near nominal syllabic rates (4.5 Hz) might have the largest impact on perception and cognition following rehabilitation. Future studies could test these possibilities.

PLV is by far the most common metric in the neuroimaging literature to compute neuroacoustic tracking of brain signals (Assaneo and Poeppel, 2018; Assaneo et al., 2019b; He et al., 2023a,b). This motivated its adoption here. In principle, both PLV and cross-correlation are methods to assess signal similarity and are in fact equivalent under a variety of circumstances (Aydore et al., 2013). One advantage of PLV is that by definition (Lachaux et al., 1999), the metric is largely invariant to amplitude scaling and depends only on phase consistency. Thus, PLV is largely impervious to fluctuations in EEG amplitude that might artificially reduce phase locking. However, PLV has been shown to be a potential biased measure under some circumstances (Aydore et al., 2013). Our data here partially confirm these concerns. We found that in the periodicity/jitter manipulation, cross-correlation largely mirrored (but was less salient than) the pattern observed for PLV (compare Fig. 5 and Extended Data Fig. 5-1). This suggests, prima facie, that mere signal correspondence may have partially driven our PLV jitter results. However, we also found cross-correlation effects were much more muted and failed to yield a differential effect (i.e., jitter × stimulus interaction) across speech/nonspeech stimuli as in PLV. An exhaustive comparison between metrics is beyond the scope of this investigation and is addressed elsewhere (Aydore et al., 2013; Soltanzadeh and Daliri, 2014). Still, the higher sensitivity and differential entrainment strength observed for PLV (but not cross-correlation) suggests the PLV metric perhaps overinflates the degree of neural phase-locked entrainment. Future studies are needed to directly assess such potential weaknesses in brain entrainment measures.

Lastly, we acknowledge the simplicity of our single-syllable tokens and the limitations of using repeated syllables to describe natural or canonical “speech” processing. While there is precedent in the literature for describing such periodic syllable trains as “speech” (Assaneo and Poeppel, 2018; Poeppel and Assaneo, 2020; He et al., 2023b), whether the differential patterns we observe for our /ba/ versus click stimuli would hold for more naturalistic (e.g., continuous and mixed-syllabic) speech remains unknown. Thus, interpretation of our findings should be limited to understanding the encoding and processing of syllable features in speech. Still, syllable approaches offer controlled and targeted investigations of specific speech features, while continuous speech approaches capture the complexity and naturalistic aspects of speech processing. Comparing these two approaches would be an interesting future direction of study.

## Synthesis

Reviewing Editor: Anne Keitel, University of Dundee Decisions are customarily a result of the Reviewing Editor and the peer reviewers coming together and discussing their recommendations until a consensus is reached. When revisions are invited, a fact-based synthesis statement explaining their decision and outlining what is needed to prepare a revision will be listed below. The following reviewer(s) agreed to reveal their identity: Christoph Daube, Christina Lubinus.

The reviewers and editor agreed that this is a potentially interesting and timely study. However, several issues were brought up by the reviewers, which are added below. In addition, the following main points were identified in joint discussions:

1) The PLV measure seems not completely appropriate to analyse the jitter conditions, because it might underestimate synchronisation at large jitters. As a case in point, reviewer #1 has written a short matlab script to demonstrate the effects of different jitters on PLV and cross-correlation (please see plvSim_eN-NWR-0027-23.m). Here are the reviewer's additional observations: "Basically, PLV indeed seems to decay for higher jitter conditions. Additionally, the question seems to be why the authors employed such a narrow band pass filter of plusminus .5 Hz. The generated signals have a broader passband. So, as far as I can see, a wider passband (matching the broader frequency range induced by the jitter) in combination with something like cross-correlation should work." In the revised manuscript, please use wider passbands and a more suitable measure for synchronisation, for example the here proposed cross-correlation.

2) The term "speech" in the current manuscript is used very loosely, as it is debatable whether the repetition of a single syllable can be compared with natural speech. This should be clearly stated from the beginning (including the abstract) and any conclusion regarding speech need to be made within the limits of the used material.

3) It would be useful to find out whether the lower PLV for clicks is due to low level stimulus properties, such as sound energy or duration. Please provide an overview of relevant properties that influence synchronisation strengths and take this into account when interpretating PLV strength.

You will find these main issues as well as other comments below. Please address all comments in a point-by-point manner.

*****

Reviewer #1 - Advances the Field (Required)

The paper provides data and results that are of relevance to a broad audience who are interested in studying the tracking of acoustic stimuli by the human brain. It provides a useful reference for questions pertaining rate and periodicity of stimulus material.

Reviewer #1 - Comments to the Authors (Required)

The authors present a study involving 24 human participants undergoing psychophysical testing as well as EEG recordings while passively listening to auditory stimuli. These stimuli vary in domain (repeated syllable "ba" vs clicks), periodicity and rate. The authors find that a form of correlation between the stimulus material and the brain responses (PLV) behaves differently across these conditions.

My favourite part of the paper was the introduction section, which provided an interesting and thorough overview over the field and succeeded in placing the present project into the wider landscape of existing research. The experimental design and analysis methods seem justified and the figures are sufficiently clear to communicate the results. However, the manuscript suffered from frustrating typos and incomplete sentences, which rendered reading unnecessarily hard.

Further, I have several points that the authors should address in order to make this manuscript publishable:

Methods:

- Stimuli: The experiment sets out to assess differences between clicks and speech. However, the "speech" used in this experiment merely consists of a single syllable "ba". This should be highlighted in all cases where this condition is referred to as the "speech" condition (I would suggest to call it e.g. "single syllable" condition). Sentences later on in the results section for example read slightly overstated given what was actually done in this experiment: Importantly, the syllable "ba" might contain much more sound energy than the very short click used, introducing an unfortunate low-level confounder in the experimental design. Ideally, the experiment would be repeated where the clicks and the syllable are matched in duration and overall sound energy integrated over time. If this is impossible, the authors should rewrite the manuscript to reflect this aspect, and devote a section of the discussion to this point.

- "Source activity was then bandpass filtered (0.9-30 Hz)" -> please provide more details of the filter (e.g. order)

- "Neural and acoustic stimulus signals were bandpass filtered ({plus minus}0.5 Hz) around each nominal frequency bin from 1.1-30 Hz" -> Was this performed on the raw stimulus signal played to the participants? At what sampling rate were these stimuli generated? Were they downsampled for this analysis? Technically, a sensible thing to do here would be to extract amplitude envelopes of the stimulus signals (e.g. abs(hilbert()) of the raw stimulus signals, then downsample). From reading the methods, I am unsure whether this was done prior to computing PLV.

Results:

- Viemeister 1979 results: I know this is a classic reference, but still I wonder: What is the significance of (if I understand correctly) measuring the threshold of a 2Hz modulation with a signal that only lasts 500ms? The caption of figure 2 explains that "TMTFs demonstrate temporal acuity for detecting AM fluctuations in continuous sounds", but the methods section [lines 129] details that these sounds only lasted 500ms, which I wouldn't call "continuous".

I would wonder if this result would look different if a longer carrier (that could fit more cycles) was used.

Lastly, could the authors add a reference that details which figure of the cited publication contains these results?

- Figure 4.: What units are the CIs computed across? Ideally, the caption would include information about how many trials / participants went into the data supporting the each data point in the plot.

- Figure 5: does the data shown here average across the "speech" and "click" conditions?

typos etc:

Abstract:

- "and jitter in ongoing sounds stream affect oscillatory entrainment" -> and jitter in ongoing sound streams affect oscillatory entrainment

- "Phase-locking to speech decreased with increasing jitter but entrainment to speech remained superior to clicks" -> I would recommend to keep the structure more parallel, i.e., do not use 2 different words within 1 sentence to refer to the same thing (phase locking and entrainment -- decide for 1 term and use it twice)

- "Surprisingly, click was invariant to periodicity manipulations" -> this could be formulated better. If taken literally, this would mean that stimuli were invariant to their manipulations, which is a paradox. I assume with "click" you mean EEG activity in the clicks condition. Please rephrase to make this more clear.

Significance Statement:

- "we tried to compare neural responses to classic psychoacoustical biomarkers of rate and periodicity sensitivity" -> did you just try to compare? Or did you actually compare? In my opinion, the whole sentence isn't needed given the previous one.

- "that speech is more sensitive to changes in rhythm and periodicity" -> do you really mean speech as being more sensitive? Or do you mean the brain in response to speech?

- line 38: "{Arnal, 2012 #24}." -> correct citation formatting

- line 48: "physiologically important behaviors" -> what are "physiologically important behaviours"? Naively, this would be something like sports?

- line 116: "approved by the Institutional Review Board at XXXX" -> replace placeholder

- line 179: "The 18 stimulus conditions" -> please add summarising information how you end up with 18 conditions as it is hard to infer that from the preceding paragraph

- line 179: "each N=1000 sweeps" -> what are sweeps here? Usually, sweeps refer to a pure tone signal changing in frequency over time

- line 211: "as this orientation capture" -> captures

- line 236: "respectively." -> here, the full stop is superscripted?

- line 266: "An ANOVA conduction on TMTF thresholds" -> remove "conduction"

- line 312: "For periodicity, we found a periodicity x stimulus interaction" -> specify what you mean by "stimulus" here -- I suppose that's "stimulus domain"?

- line 339: "For rate, we found phase-locking to" -> we found that phase-locking [...]

- line 357: "We found PLV strongly differentiated speech vs. non-speech stimuli" -> sentence incomplete

- line 362: "synrhonization" -> synchronization

- line 385: "where adaptation would play an effect." -> rephrase

- line 402: following: "Our PLV analysis of cortical entrainment at this rate demonstrate periodicity did not affect across-token phase locking to clicks" -> rewrite sentence

- line 422: "exhibit less robustness between token measures." -> don't understand what is referred to here with "robustness between token measures"

- line 463: "Our finding that speech is particularly rate and periodicity sensitive" -> how can speech be sensitive? Rethink the subject of this phrase.

*****

Reviewer #2 - Advances the Field (Required)

The paper investigates how auditory tracking is influenced as speech and non-speech signals are manipulated with respect to periodicity (and rate). Even though it is common knowledge that speech is a quasi-rhythmic signal at best, the larger speech tracking/entrainment community assumes/treats speech and brain signals as oscillatory for feasibility reasons (many methods assume the input variables to be oscillatory). Therefore, the paper sets out to investigate an important potential confound in the field. However, even if the question is relevant, I am unsure whether the results in their current state provide useful insights due to some methodological details.

Reviewer #2 - Statistics

The statistics (Anova and correlations) seem sound. However, the assumptions for the speech tracking analysis with the current measure may be violated. I suggest repeating the analysis with a measure that is not sensitive to aperiodicity.

Reviewer #2 - Comments to the Authors (Required)

Summary:

The paper examines the effect of a parametric manipulation of the rate and periodicity of syllable and click trains on the tracking of the stimulus in auditory cortex. Participants performed a passive-listening paradigm during EEG recording and the authors employed source localization and phase-locking value analyses to quantify auditory "entrainment"/tracking.

The study reports enhanced speech tracking at 4.5Hz with lower brain-to-stimulus synchronization for lower and faster syllabic rates. In contrast, for click sequences tracking was lowest at 4.5Hz but increased for both faster and slower rates. With respect to periodicity, an interaction of periodicity and stimulus domain (speech vs clicks) is reported, with higher PLVs for speech stimuli in all but one jitter condition. Finally, the correlation of PLV (averaged across rates) and a behavioral measure (TMTF thresholds, collected prior to EEG experiment) revealed an effect, suggesting stronger tracking in individuals with poorer (i.e. less negative) TMTF thresholds. The authors claim that the brain may prioritize speech as compared to other auditory stimuli, as evidenced by the enhanced tracking of the speech signals.

General evaluation:

The study sets out to answer a timely question The paper could use editing to address language issues and typos but the main points are communicated well. Overall, I have two major concerns:

Firstly, while the PLV analysis is well-established for (quasi-)periodic signals (e.g. here the rate condition where jitter is 0%), I wonder whether this measure -being sensitive to aperiodicity in signals- is the best method to assess the effect of periodicity on entrainment.

Secondly, entrainment of the auditory cortex to non-speech sounds such as clicks or noise has been reported widely, particularly for low frequencies. Therefore, I was surprised by the low PLVs at 4.5Hz and, more generally, by the overall lower tracking of the click trains.

Major issues

PLV:

My first main concern refers to the PLV as a measure to quantify entrainment in an aperiodic signal. From my understanding, this measure is used widely to quantify speech tracking so that I assume, a certain degree of aperiodicity can be tolerated (given that speech is only quasi-periodic). However, given that periodicity was actively (and strongly) disrupted in the periodicity condition -and knowing that the PLV is not defined for non-isochronous signals, strictly speaking- I wonder if the PLV can reflect the truthful degree of synchronization between stimulus and auditory cortex in this condition at all. I would be more convinced if the same pattern of results could be replicated using a measure that is not as sensitive to aperiodicity.

Clicks:

Entrainment/tracking of complex sounds such as speech and music is strongest in low frequencies. This is in line with the findings reported in the speech condition of the current study. However, entrainment to low-frequency stimuli has not only been observed for complex sounds (e.g. speech and music) but also for more low-level sounds such as amplitude modulated noise etc. In contrast to this, the study reports low PLVs for the click train condition at low frequencies, the minimum PLV being located at 4.5 Hz. Would one not have expected equal PLVs for the click as compared to the speech condition at this rate? Do the authors think that this may be related to the difference in sound duration (100μs vs 50ms for clicks vs speech, respectively)?

Minor issues

Methods/results lack details

- l. 179: The authors state that overall, the EEG experiment comprised 18 conditions. However, it is unclear how these conditions come about and, importantly, what they entail (how long are the stimuli? How many are there?). From Figure 2 it seems that each condition contained many trials but the exact number of trials per condition is not stated anywhere.

- l. 322: With respect to the brain-behavior correlations, two aspects were unclear to me:

1. It is stated that the PLV was collapsed across stimulus rates. However, was the PLV also collapsed across both stimulus modalities (speech and clicks) or was the correlation computed for the PLVs of the speech and click conditions separately - and if so which results are being reported and visualized in Figure 5?

2. From Figure 5 and the description of the results for the correlation of CA-BAT and PLVs, I assume that multiple correlations of PLV and TMTF threshold were computed (for the PLV at each rate plus the mean-PLV). However, only the result for the correlation of mean-PLV and TMTF thresholds is reported. For completeness, I would suggest reporting all results.

l. 191: Silent movie watching during passive listening seems somewhat untypical for the speech tracking literature. Could you please elaborate on your motivation for this choice of paradigm?

l. 238: My understanding is that the PLV between stimulus and source-localized activity was not only computed at the stimulation rate but across a wide range of frequencies. These results are visualized in Figure 3. What I would have expected is a dominant peak around the stimulation frequency with significantly lower PLVs for all other frequencies (as can be seen for the 8.5 and 14.9 Hz conditions). However, for the slower rates (</= 4.5 Hz) many prominent peaks are visible. Could you elaborate why you think you observed such an oscillatory-looking pattern in the slower rate conditions?

l. 242: To compare PLVs across rates, the "peak" PLV around stimulation rate (+/- 0.5Hz) was extracted. Prior to extracting the peak value, was there a check if the defined window actually contained a peak in or was it simply the maximal PLV that was extracted? In other words, did the extracted value reflect the most prominent peak (or even a peak at all)? It might be possible that the synchronization did in fact not occur around stimulation rate but at a shifted frequency?

l. 437: The authors reported a correlation of mean-PLV (averaged across rates) and TMTF and interpret the link to reflect effort of encoding. If the tracking in fact reflected effort, would one not expect to see a modulation across stimulation rates? I.e. stronger correlation for faster frequencies (which should be more effortful to encode)?

## Author Response

Response to Reviewers We thank the reviewers for their thoughtful comments and suggestions and the opportunity to revise our manuscript. In this revision, we address these concerns to the best of our ability. These changes have greatly improved the quality of the manuscript. Changes are summarized below and appear in red font in the main text.

Editor Synthesis of Reviews:

E1 The PLV measure seems not completely appropriate to analyse the jitter conditions because it might underestimate synchronisation at large jitters. As a case in point, reviewer #1 has written a short matlab script to demonstrate the effects of different jitters on PLV and cross-correlation (please see plvSim_eN-NWR-0027-23.m). Here are the reviewer's additional observations: "Basically, PLV indeed seems to decay for higher jitter conditions. Additionally, the question seems to be why the authors employed such a narrow band pass filter of plusminus .5 Hz. The generated signals have a broader passband. So, as far as I can see, a wider passband (matching the broader frequency range induced by the jitter) in combination with something like cross-correlation should work." In the revised manuscript, please use wider passbands and a more suitable measure for synchronisation, for example the here proposed cross-correlation.

Response: We appreciate the reviewer's perspective and the provided MATLAB code to demonstrate the (acoustic) effects of different jitters on PLV and cross-correlation. In principle, both PLV and cross-correlation are methods to assess signal similarity and are in fact equivalent under a variety of circumstances (Aydore et al., NeuroImage, 2013). As the reviewer's toy example nicely illustrates, they also produce the same pattern of results as reported in our paper, so the choice of the particular metric is largely inconsequential. Our selection of PLV is twofold. First, PLV is the most commonly used method in the human EEG literature to assess neural synchronization, including seminal studies examining the neural entrainment to speech (Assaneo et al., Nat Neuro, 2019; Doelling et al, PNAS, 2019; Assaneo & Poeppel, Science Adv., 2018). Second, our own recent studies examining auditory-brain speech synchronization have employed PLV. Thus, we feel it pragmatic to reuse this metric in the current study to allow direct comparison and replication of published work.

To be reasonably interpreted, PLV (cross-correlation too) requires first bandpass filtering the signals to assess how entertainment changes in a frequency-dependent manner (as in Fig. 4). In our approach, adopted from Assaneo & Poeppel (2018) and He et al. (2018), the signals are first bandpass filtered in a 1 Hz band around the nominal stimulus rate and PLV was computed. This is then repeated for a range of center frequencies from 0.9-30 Hz to track the relative change in PLV across rates, and jitter manipulations. These points have been clarified in the methods. Interestingly, our results did not show a significant decline in PLV for increasing jitter in the click stimulus. However, we did observe general desynchronization when applying jitter to "ba" stimuli. These contrasting outcomes between different stimulus domains confirm that while PLV is not the sole measure of sound-to-brain synchronization, it nevertheless captures important stimulus-specific properties of entrainment.

E2 The term "speech" in the current manuscript is used very loosely, as it is debatable whether the repetition of a single syllable can be compared with natural speech. This should be made clear from the beginning (including the abstract) and conclusions regarding speech need to be made within the limits of the used material.

Response: We agree and have toned down the interpretation of our syllable train stimuli as natural "speech." We now qualify with terms like "speech-like stimuli" (abstract) and throughout. However, we note the precedent to describe repeated syllable trains as "speech" is common in many speech-tracking studies (Assaneo et al, 2018; 2019; Doelling et al, 2019). Still, we agree it is important to clarify the limitations of using single syllables and acknowledge the debate surrounding their comparison to natural speech. This is added now as a future direction in last paragraph of conclusion: "Lastly, we acknowledge the simplicity of our single syllable tokens and the limitations of using repeated syllables to describe natural/canonical "speech" processing. While there is precedent in the literature for describing such periodic syllable trains as "speech", whether or not the differential patterns we observe for our /da/ vs. click stimuli would hold for more naturalistic (e.g., continuous and multi-syllabic) speech remains unknown..." E3 It would be useful to find out whether the lower PLV for clicks is due to low level stimulus properties, such as sound energy or duration. Please provide an overview of relevant properties that influence synchronisation strengths and take this into account when interpreting differences between conditions.

Response: We appreciate this suggestion. We have incorporated a new figure (Figure 1) of the revised manuscript which shows the level and bandwidth of the isolate tokens was well matched. Level was also equated (74 dB SPL) for both speech and non-speech stimuli.

RESPONSE TO REVIEWER #1:

R1.1 The paper provides data and results that are of relevance to a broad audience who are interested in studying the tracking of acoustic stimuli by the human brain. It provides a useful reference for questions pertaining rate and periodicity of stimulus material.

Response: Thank you for your kind appraisal of our work and opportunity to address your concerns in a revision.

R1.2 The manuscript suffered from frustrating typos and incomplete sentences, which rendered reading unnecessarily hard.

Response: We apologize for the typos and incomplete sentences in the initial manuscript. In the current version, we have made significant efforts to address these issues and improve the overall flow of the text.

R1.3 Stimuli: The experiment sets out to assess differences between clicks and speech. However, the "speech" used in this experiment merely consists of a single syllable "ba". This should be highlighted in all cases where this condition is referred to as the "speech" condition (I would suggest to call it e.g. "single syllable" condition). Sentences later on in the results section for example read slightly overstated given what was actually done in this experiment: Importantly, the syllable "ba" might contain much more sound energy than the very short click used, introducing an unfortunate low-level confounder in the experimental design. Ideally, the experiment would be repeated where the clicks and the syllable are matched in duration and overall sound energy integrated over time. If this is impossible, the authors should rewrite the manuscript to reflect this aspect, and devote a section of the discussion to this point.

Response: We agree that using the term "single syllable" is perhaps more accurate than "speech." We have attempted to qualify the term "speech" throughout and also added this point as a limitation (p.19). See also response to E3.

Regarding the sound energy, we apologize for any confusion caused by our previous statement. Both the click and the syllable "ba" stimulus trains were indeed calibrated to have the same level. Thus, overall intensity effects can be ruled out. Level effects would also manifest in a parallel pattern across speech and non-speech stimuli and this is not what we observe (Fig. 5). We have also added a new Fig. 1. showing the overall bandwidth of the stimuli were matched. Signal duration of course differs between a transient click and sustained speech token. See also response to R2.3 for additional edits on this point.

R1.4 - "Source activity was then bandpass filtered (0.9-30 Hz)" -> please provide more details of the filter (e.g. order) Response: We now clarify the filter was a 10th order Butterworth.

R1.5 "Neural and acoustic stimulus signals were bandpass filtered ({plus minus}0.5 Hz) around each nominal frequency bin from 1.1-30 Hz" -> Was this performed on the raw stimulus signal played to the participants? At what sampling rate were these stimuli generated? Were they downsampled for this analysis? Technically, a sensible thing to do here would be to extract amplitude envelopes of the stimulus signals (e.g. abs(hilbert()) of the raw stimulus signals, then downsample). From reading the methods, I am unsure whether this was done prior to computing PLV.

Response: We have clarified in the methods that acoustic stimuli were presented in the listening task at a sampling rate of 48818 Hz to ensure maximal acoustic fidelity. However, stimuli were later downsampled in the analysis to match the sampling rate of the EEG. Because we are only interested in the band limited, low-frequency syllable rate envelope, filtering was performed prior to PLV calculations.

R1.6 Viemeister 1979 results: I know this is a classic reference, but still I wonder: What is the significance of (if I understand correctly) measuring the threshold of a 2Hz modulation with a signal that only lasts 500ms? The caption of figure 2 explains that "TMTFs demonstrate temporal acuity for detecting AM fluctuations in continuous sounds", but the methods section [lines 129] details that these sounds only lasted 500ms, which I wouldn't call "continuous". I would wonder if this result would look different if a longer carrier (that could fit more cycles) was used.

Response: We have deleted "continuous" to avoid confusion. Our use of the 500 ms carrier was to replicate the gated-carrier paradigm in Viemeister (1979) (their Fig. 6). As the reviewer points out, these means that for the lowest f¬m, subjects only heard part of the cycle of the modulation. However, in the 2AFC task, the fluctuation is still quite perceptually salient. In general, detection improves slightly with duration, consistent with the longer carrier providing more "looks" to detect the AM signal (Almishaal, Bidelman, Jennings, 2017; Viemeister, 1979).

R1.7 Lastly, could the authors add a reference that details which figure of the cited publication contains these results? Response: Reference added to their Fig. 6 (500 ms gated carrier condition).

R1. 8 Figure 4: What units are the CIs computed across? Ideally, the caption would include information about how many trials / participants went into the data supporting the each data point in the plot.

Response: We now clarify that the traces reflect the grand average across subjects as well as the units for the variance shading (= {plus minus}1 s.e.m.) R1.9 Figure 5: does the data shown here average across the "speech" and "click" conditions? Response: Correct. We now specify the scatter figures represent pooled data across both the "speech" and "click" conditions.

R1.10 Typos etc:

Abstract:

- "and jitter in ongoing sounds stream affect oscillatory entrainment" -> and jitter in ongoing sound streams affect oscillatory entrainment - "Phase-locking to speech decreased with increasing jitter but entrainment to speech remained superior to clicks" -> I would recommend to keep the structure more parallel, i.e., do not use 2 different words within 1 sentence to refer to the same thing (phase locking and entrainment -- decide for 1 term and use it twice) - "Surprisingly, click was invariant to periodicity manipulations" -> this could be formulated better. If taken literally, this would mean that stimuli were invariant to their manipulations, which is a paradox. I assume with "click" you mean EEG activity in the clicks condition. Please rephrase to make this more clear.

Response: Thank you for your feedback. We modified these statements for clarification.

R1.10 Significance Statement:

- "we tried to compare neural responses to classic psychoacoustical biomarkers of rate and periodicity sensitivity" -> did you just try to compare? Or did you actually compare? In my opinion, the whole sentence isn't needed given the previous one.

- "that speech is more sensitive to changes in rhythm and periodicity" -> do you really mean speech as being more sensitive? Or do you mean the brain in response to speech? - line 38: "{Arnal, 2012 #24}." -> correct citation formatting - line 48: "physiologically important behaviors" -> what are "physiologically important behaviours"? Naively, this would be something like sports? - line 116: "approved by the Institutional Review Board at XXXX" -> replace placeholder - line 179: "The 18 stimulus conditions" -> please add summarising information how you end up with 18 conditions as it is hard to infer that from the preceding paragraph - line 179: "each N=1000 sweeps" -> what are sweeps here? Usually, sweeps refer to a pure tone signal changing in frequency over time - line 211: "as this orientation capture" -> captures - line 236: "respectively." -> here, the full stop is superscripted? - line 266: "An ANOVA conduction on TMTF thresholds" -> remove "conduction" - line 312: "For periodicity, we found a periodicity x stimulus interaction" -> specify what you mean by "stimulus" here -- I suppose that's "stimulus domain"? - line 339: "For rate, we found phase-locking to" -> we found that phase-locking [...] - line 357: "We found PLV strongly differentiated speech vs. non-speech stimuli" -> sentence incomplete - line 362: "synrhonization" -> synchronization - line 385: "where adaptation would play an effect." -> rephrase - line 402: following: "Our PLV analysis of cortical entrainment at this rate demonstrate periodicity did not affect across-token phase locking to clicks" -> rewrite sentence - line 422: "exhibit less robustness between token measures." -> don't understand what is referred to here with "robustness between token measures" - line 463: "Our finding that speech is particularly rate and periodicity sensitive" -> how can speech be sensitive? Rethink the subject of this phrase.

Response: These edits have been made at their respective places in the text.

RESPONSE TO REVIEWER #2:

R2.1 Advances the Field (Required). The paper investigates how auditory tracking is influenced as speech and non-speech signals are manipulated with respect to periodicity (and rate). Even though it is common knowledge that speech is a quasi-rhythmic signal at best, the larger speech tracking/entrainment community assumes/treats speech and brain signals as oscillatory for feasibility reasons (many methods assume the input variables to be oscillatory). Therefore, the paper sets out to investigate an important potential confound in the field. However, even if the question is relevant, I am unsure whether the results in their current state provide useful insights due to some methodological details.

Response: We would like to express our gratitude to the reviewer for their time and attention to our manuscript. We understand their concerns and appreciate their recognition of the relevance of the research question.

R2.2 Statistics: The statistics (Anova and correlations) seem sound. However, the assumptions for the speech tracking analysis with the current measure may be violated. I suggest repeating the analysis with a measure that is not sensitive to aperiodicity. Overall, I have two major concerns: PLV: Firstly, while the PLV analysis is well-established for (quasi-)periodic signals (e.g. here the rate condition where jitter is 0%), I wonder whether this measure -being sensitive to aperiodicity in signals- is the best method to assess the effect of periodicity on entrainment. My first main concern refers to the PLV as a measure to quantify entrainment in an aperiodic signal. From my understanding, this measure is used widely to quantify speech tracking so that I assume, a certain degree of aperiodicity can be tolerated (given that speech is only quasi-periodic). However, given that periodicity was actively (and strongly) disrupted in the periodicity condition -and knowing that the PLV is not defined for non-isochronous signals, strictly speaking- I wonder if the PLV can reflect the truthful degree of synchronization between stimulus and auditory cortex in this condition at all. I would be more convinced if the same pattern of results could be replicated using a measure that is not as sensitive to aperiodicity.

Response: Please see response to E2. We appreciate the reviewer's perspective and the provided MATLAB code to demonstrate the (acoustic) effects of different jitters on PLV and cross-correlation. In principle, both PLV and cross-correlation are methods to assess signal similarity and are in fact equivalent under a variety of circumstances (Aydore et al., NeuroImage, 2013). As the reviewer's toy example nicely illustrates, they also produce the same pattern of results as reported in our paper, so the choice of the particular metric is largely inconsequential. Our choice of using PLV is twofold. First, PLV is the most commonly used method in the human EEG literature to assess neural synchronization and in seminal studies examining the neural entrainment to speech (Assaneo et al., Nat Neuro, 2019; Doelling et al, PNAS, 2019; Assaneo & Poeppel, Science Adv., 2018). Second, our own recent studies examining auditory-brain speech synchronization have employed PLV so we feel it pragmatic to reuse in this metric in the current study to allow for direct comparison and replication of published work.

R2.3 Clicks: Secondly, entrainment of the auditory cortex to non-speech sounds such as clicks or noise has been reported widely, particularly for low frequencies. Therefore, I was surprised by the low PLVs at 4.5Hz and, more generally, by the overall lower tracking of the click trains. Entrainment/tracking of complex sounds such as speech and music is strongest in low frequencies. This is in line with the findings reported in the speech condition of the current study. However, entrainment to low-frequency stimuli has not only been observed for complex sounds (e.g. speech and music) but also for more low-level sounds such as amplitude modulated noise etc. In contrast to this, the study reports low PLVs for the click train condition at low frequencies, the minimum PLV being located at 4.5 Hz. Would one not have expected equal PLVs for the click as compared to the speech condition at this rate? Do the authors think that this may be related to the difference in sound duration (100μs vs 50ms for clicks vs speech, respectively)? Response: Your observation regarding the lower PLV and overall lower tracking of click trains compared to speech stimuli at low frequencies was also unexpected for us. While it is true that the overall nature of tracking is lowpass in nature, a more general look across lower rates actually reveals a bandpass-like shape, with enhancement near 4-5Hz, the nominal rate of speech in most languages. This "resonance" has been independently reported in several studies on speech-brain entrainment from different labs (He et al., 2023; Assaneo & Poeppel, 2018). See also response to E2.

The lower PLV observed in the click, specifically the trough at 4.5 Hz, was also unexpected. Duration of the stimuli is one possible factor that could contribute to the difference in PLV between the two stimulus domains. But is important to note the main effect the reviewer refers too (i.e., PLVspeech > PLVclick) is misleading in light of the interaction observed between speech and click stimuli (Fig. 5A). But it is difficult to see how duration effects alone could account for the differential (interaction) pattern observed in our data between speech and click trains. There is some evidence that auditory cortical entrainment is enhanced for intelligible signals which might explain the larger PLV we see for the syllable train relative to click stimuli (Xu et al., 2021). Moreover, token duration seems to play a less prominent role in the strength of cortical entrainment since tracking is dominated by locking to an acoustic signal's edge landmarks rather than its nucleus, per se (Oganian et al., 2022; Oganian & Chang, 2019). These points have been added to the discussion.

R2.4 Minor issues Methods/results lack details - l. 179: The authors state that overall, the EEG experiment comprised 18 conditions. However, it is unclear how these conditions come about and, importantly, what they entail (how long are the stimuli? How many are there?). From Figure 2 it seems that each condition contained many trials but the exact number of trials per condition is not stated anywhere.

Response: We have clarified: "The study encompassed both speech and click conditions, involving a total of five rates (2.1, 3.3, 4.5, 8.5, 14.9 Hz) and five jitter conditions specifically applied at the 4.5 Hz rate (0, 20, 40, 60, 80%). In total, there were 18 distinct conditions (the 0% jitter was the same in the 4.5 Hz rate). Each condition consisted of 1000 tokens". This is now added on page 7.

R2.5 l. 322: With respect to the brain-behavior correlations, two aspects were unclear to me:

1. It is stated that the PLV was collapsed across stimulus rates. However, was the PLV also collapsed across both stimulus modalities (speech and clicks) or was the correlation computed for the PLVs of the speech and click conditions separately - and if so which results are being reported and visualized in Figure 5? 2. From Figure 5 and the description of the results for the correlation of CA-BAT and PLVs, I assume that multiple correlations of PLV and TMTF threshold were computed (for the PLV at each rate plus the mean-PLV). However, only the result for the correlation of mean-PLV and TMTF thresholds is reported. For completeness, I would suggest reporting all results.

Response: In the mean condition shown in Fig. 6, we averaged all five rates. All panels also collapse PLV across both /da/ and click stimulus modalities to reduce the dimensionality of the data. As suggested, we now also report separate correlations shown in Fig. 6 in the text on page 13.

R2.6 l. 191: Silent movie watching during passive listening seems somewhat untypical for the speech tracking literature. Could you please elaborate on your motivation for this choice of paradigm? Response: During passive EEG recording, one common technique to maintain participant engagement while minimizing movements and potential artifacts is to have them watch a silent movie. This means that in our study, participants were not actively engaged in a specific task or focusing their attention on the auditory stimuli. However, passive listening was an intended part of our design. As discussed on p. 15, the fact that we still find enhanced entrainment at 4-5 Hz confirms that the putative language-dependent tuning of the speech entrainment system to these select frequencies (e.g., Assaeno & Poeppel, 2018; He et al., 2023) does not depend on attention but is automatic, which is a novel finding. This justification has also been added to the methods: "Passive listening allows for the investigation of spontaneous neural responses without the influence of specific cognitive or attentional demands. Here, it was used explicitly used to test whether previously reported enhancements in neural speech tracking near the language-universal syllable rate (4-5 Hz) depend on attention or instead reflect more automatic tuning of auditory entrainment processes".

R2.7 l. 238: My understanding is that the PLV between stimulus and source-localized activity was not only computed at the stimulation rate but across a wide range of frequencies. These results are visualized in Figure 3. What I would have expected is a dominant peak around the stimulation frequency with significantly lower PLVs for all other frequencies (as can be seen for the 8.5 and 14.9 Hz conditions). However, for the slower rates (Response: Correct. PLV was computed across a range (1.1-30 Hz) of frequencies, creating a continuous function of entrainment strength across frequencies (Fig. 4). As noted in the figures caption, the higher frequency peaks the reviewer refers to are harmonics of the fundamental rate. Harmonics are common in the spectra of sustained auditory potentials due to nonlinearities in the EEG, resulting in phase locking at the F0 and its integer multiples (2F0, 3F0, etc.). This point is now mentioned on p.12. They appear more frequent for the lower rates because there are more harmonics in our 30 Hz analysis bandwidth window; the higher 14.9 Hz rate would show a harmonic at 29.8 Hz but is outside the plotting window.

R2.8 l. 242: To compare PLVs across rates, the "peak" PLV around stimulation rate (+/- 0.5Hz) was extracted. Prior to extracting the peak value, was there a check if the defined window actually contained a peak in or was it simply the maximal PLV that was extracted? In other words, did the extracted value reflect the most prominent peak (or even a peak at all)? It might be possible that the synchronization did in fact not occur around stimulation rate but at a shifted frequency? Response: Nearly all subjects showed peaks within 0.5 Hz of the nominal rate. We now report mean{plus minus}STDs for the peak PLV location on p.12: "In general, neural responses closely followed the speed of the auditory stimuli, showing stark increases in PLV at that closely followed the fundamental rate of presentation (pooling across hemispheres, 2.1 Hz=2.4{plus minus}0.2 Hz; 3.3 Hz=3.5{plus minus}0.3 Hz; 4.5 Hz=4.4{plus minus}0.3 Hz; 8.5 Hz= 8.5{plus minus}0.3 Hz; 14.9 Hz=15.0{plus minus}0.3 Hz)." R2.9 l. 437: The authors reported a correlation of mean-PLV (averaged across rates) and TMTF and interpret the link to reflect effort of encoding. If the tracking in fact reflected effort, would one not expect to see a modulation across stimulation rates? I.e. stronger correlation for faster frequencies (which should be more effortful to encode)? Response: If mean PLV averaged across rates indeed reflects the effort of encoding, it would be reasonable to expect a modulation in the correlation across stimulation rates. The reasoning behind this expectation is that faster frequencies typically involve rapid temporal changes and higher information processing demands. Participants may need to allocate more cognitive resources and exert greater effort to accurately track and encode stimuli presented at faster rates. However, we agree this point is speculative given the passive nature of our task and generally weak correlations in our data. We now qualify this in the discussion (p.19): '"Consequently, given our strictly passive listening paradigms, interpretations that our brain-behavioral correlations index listening effort remains speculative and should be confirmed in future studies."
