# Supplementary figures and images for: Effects of Stimulus Rate and Periodicity on Auditory Cortical Entrainment to Continuous Sounds
Source: eNeuro. 2024 Mar 1;11(3):ENEURO.0027-23.2024. doi: 10.1523/ENEURO.0027-23.2024 (PMC10913036; doi:10.1523/ENEURO.0027-23.2024)

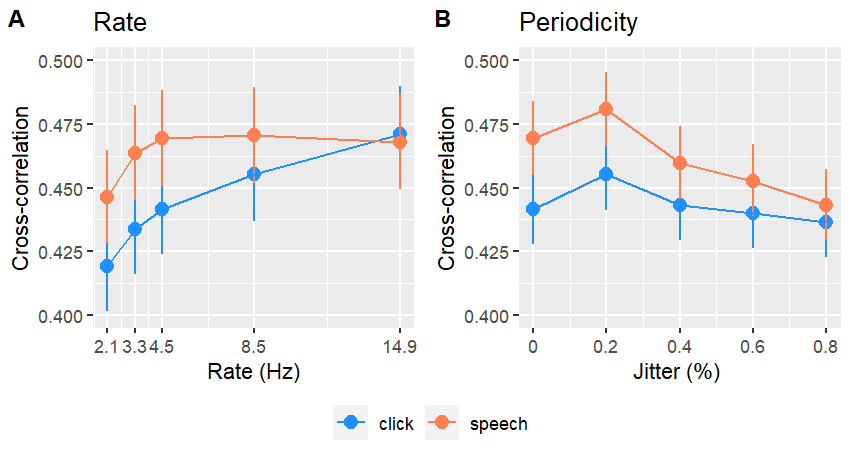

Supplement: Figure 5-1 — Grand average peak stimulus-to-EEG cross-correlation across (A) rate and (B) jitter aperiodicity. Cross-correlations highly differ from the PLV pattern observed for rate. They partially replicate the PLV analysis in Fig. 5 for jitter but do not show a jitter* stimulus interaction like PLV. Errorbars denote 95% CIs. Download Figure 5-1, TIF file. [file eneuro-11-ENEURO.0027-23.2024-s001.tif]
